# Healthcare workforce transformation: implementing patient-centered medical home standards in an academic medical center

**DOI:** 10.1186/s12909-021-02775-9

**Published:** 2021-06-03

**Authors:** Rebecca Gendelman, Heidi Preis, Latha Chandran, Robyn J. Blair, Maribeth Chitkara, Susmita Pati

**Affiliations:** 1 Renaissance School of Medicine, Stony Brook University, NY Stony Brook, USA; 2 Department of Psychology, Stony Brook University, NY Stony Brook, USA; 3 Department of Pediatrics, Renaissance School of Medicine, Stony Brook University, Health Sciences Center Level 11, Suite 20, NY 11794-8111 Stony Brook, USA

**Keywords:** Medical home, Change management, Medical education, Healthcare workforce training

## Abstract

**Background:**

Large scale implementation of new strategies and healthcare delivery standards in academic medical centers (AMCs) requires training of healthcare workforce at different stages of their medical career. The patient-centered medical home (PCMH) model for healthcare delivery involves adoption by all members of the healthcare workforce, including seasoned professionals and trainees. Though widely known, the PCMH model has been implemented sporadically at large AMCs and methods to implement the model across healthcare workforce have not been well-documented.

**Methods:**

To meet all PCMH standards and achieve sustainable level 3 recognition, the authors implemented in 2014–2015 a multi-pronged approach that capitalized on existing educational infrastructure among faculty, residents, and medical students. Within 18 months, the authors applied new interdisciplinary practices and policies, redesigned residency training in continuity practices and extensively modified medical school curricula.

**Results:**

These innovative transformational education efforts addressed the six PCMH standards for faculty, residents, and undergraduate medical students. Faculty played a major role as system change agents and facilitators of learning. Residents learned to better understand patients’ cultural needs, identify ‘at-risk’ patients, ensure continuity of care, and assess and improve quality of care. Medical students were exposed to PCMH core standards throughout their training via simulations, training in the community and with patients, and evaluation tasks. By implementing these changes across the healthcare workforce, the AMC achieved PCMH status in a short time, changed practice culture and improved care for patients and the community. Since then, the AMC has been able to maintain PCMH recognition annually with minimal effort.

**Conclusions:**

Successful strategies that capitalize on existing strengths in infrastructure complemented by innovative educational offerings and inter-professional partnerships can be adapted by other organizations pursuing similar transformation efforts. This widespread transformation across the healthcare workforce facilitate a deep-rooted change that enabled our academic medical center to sustain PCMH recognition.

**Supplementary Information:**

The online version contains supplementary material available at 10.1186/s12909-021-02775-9.

## Background

Successful integration of patient-centered principles into care requires changes in practice, knowledge, attitudes and policies across the entire healthcare workforce [1]. The magnitude and scale of this change management effort has made implementation of the patient-centered medical home (PCMH) model in Academic Medical Centers (AMC) challenging [2, 3]. The PCMH model is widely promoted to support high quality primary care by leveraging interdisciplinary teamwork in practice [4]. Originally, the development and conceptualization of the medical home came from the field of pediatrics through work sponsored by the American Academy of Pediatrics and the Maternal and Child Health Bureau [5]. The National Committee for Quality Assurance (NCQA) promulgated and codified these concepts and core principles into a set of standards that are evaluated to award PCMH recognition to practices, which enables collection of enhanced reimbursements from some public and private payors [6]. These incentives have spurred a variety of innovations in graduate and undergraduate medical education– predominantly in the fields of Family Medicine and Internal Medicine [3, 7]—but little has been done for faculty. Since PCMH metrics regularly evolve and many faculty are relative novices in this area, the classic linear educational model is no longer applicable and requires faculty to learn concurrently with the entire healthcare workforce including trainees [8, 9]. Despite the importance of thorough implementation to achieve optimal outcomes of PCMH, including reduced expenditure,[10] little has been published about real-world PCMH implementation efforts across the healthcare workforce. In this paper we describe our institution’s practice transformation educational efforts to integrate the PCMH model and its principles into medical education across three key groups comprising the AMC physician workforce– medical students, residents, and faculty.

## Methods

Various Quality Improvement (QI) data were compiled to achieve PCMH Level 3 recognition from NCQA that were used in the current report. These include chart reviews, audits, and routine reports from our multiple data systems (i.e. administrative billing data, clinical quality, etc.) to evaluate our efforts. Additionally, to evaluate our efforts, we reviewed minutes from faculty and staff meetings, documentation of curricular revisions, and gathered input from the authors (RJB is the Pediatric Residency Program Director and Vice Chair for Education, MBC is the Director of the Medical Student Education, LC is the former Vice Dean for Undergraduate Medical Education, and SP is the Division Chief for Primary Care Pediatrics and led these PCMH transformation efforts for Stony Brook Medicine in her role as Medical Director of the Hospital-Medical Home Demonstration Initiative) [11]. This initiative was funded by the Centers for Medicaid and Medicare Services and administered by the New York State Department of Health. Our institution was required to meet all six PCMH standards at all primary care continuity training sites (i.e., 9 sites with > 150 healthcare workforce staff; 5 of 9 sites served as resident continuity practice locations and all sites served as potential medical student training locations) within an 18-month period beginning in 2014–2015 and subsequently sustain these efforts to maintain recognition basis. Since PCMH recognition was required by all primary continuity clinic training sites across three departments (Family Medicine, Internal Medicine, and Pediatrics), faculty and trainees at different stages of training were included in our transformation efforts and lessons learned were shared between departments. Though we describe specific examples from our work within the department of pediatrics in this report, many of these approaches were implemented across all three departments under Dr. Pati’s leadership [12]. Educational outcomes were not measured for this project.

Successful implementation of PCMH requires coordination and training of a highly diversified and large workforce [1, 8, 9] and we utilized change management principles and drew upon the literature about organizational behavior to undertake this transformation [2–4, 6, 13–16]. In particular, our implementation strategy paid careful attention to the fact that each individual health care professional – whether faculty or trainee– has limited hours available for educational activities. Taking this practical limitation into account and as others have recommended, [17] we integrated our PCMH training into existing continuing education opportunities (e.g., faculty and resident retreats, nursing recertification programs, etc.) as well as graduate and undergraduate medical education offerings (e.g., courses, lecture series, etc.). This allowed for staff and trainees alike to learn without further burdening already busy schedules. Notably, our organization implemented electronic medical record (EMR) adoption (Cerner PowerChart [18]) at the same time as we pursued our initial PCMH recognition, a practice which is pivotal in adapting team-based primary care[19]; we partnered closely with our information technology staff to create documentation and workflow plans that would support PCMH recognition as efficiently as possible by leveraging EMR functions such as auto-texts and order sets.

To launch this effort, we conducted a full-day retreat for all ambulatory practice staff facilitated by an extramural consultant to introduce PCMH core tenets and the corresponding NCQA standards. Subsequently, with guidance from our extramural consultant and patient-centered medical home coordinator (holding dual master’s degrees in economics and computer science), policies and procedures addressing the PCMH standards were initially written (during a 3-hour in-person meeting), reviewed, and disseminated by an interdisciplinary leadership team comprised of the department chairperson and administrator, the primary care division chief, and the ambulatory nursing supervisor with input from our information technology team as needed. Thereafter, we organized the residency curriculum to include the core NCQA PCMH standards in the ambulatory resident continuity practice setting (learning session every 6 weeks) and in the routine resident lecture series. In the summer of 2014, our School of Medicine launched a completely revised curriculum in an effort to create system-based courses with enhanced active learning and a renewed focus on patient-centeredness. The LEARN (Learner-entered, Experiential, Adaptive, Rigorous and Novel) curriculum incorporated the NCQA PCMH standards into undergraduate medical education, both in the pre-clinical and clinical phases. Institutional Learning Objectives were mapped to all courses, pre-clinical and clinical, to ensure that each objective would be addressed at specific timepoints during undergraduate medical education.

In the ensuing sections, we describe our transformation and sustainability efforts for each of the PCMH NCQA standards across the healthcare workforce. Detailed comparisons of the former and transformed states can be found in Tables 1 and 2, and 3 for faculty, residents and students respectively.

**Table 1:** Faculty development: Strategies addressing PCMH principles pre- and post-transformation

Former State	Current State
**1. Team-Based Care**	
• Team members (i.e. faculty, nurses, and ancillary staff) at each site worked together ad hoc to complete a broad array of patient care tasks. • All team members expected to role-model culturally competent practice skills by caring for a diverse patient population.	• Clearly defined roles for all team members: In particular, practice reception staff are responsible for demographic intake, nursing staff are responsible for managing patient throughout, and faculty are responsible for running daily huddles, directing practice staff, and identifying patients for care management. • Each practice site has a designated faculty physician lead, clinical nursing lead, and office manager that meet formally every month to discuss ways to optimize practice operations. • Faculty mentor residents to lead daily huddles and review pre-visit planning during continuity clinic.
**2. Patient-Centered Access**	
• Patients required to follow cumbersome process to request medical records, including vaccination history and test results. • Patients asked to specify provider when calling to make an appointment. Patient requests for same day sick appointments were managed by individual providers. • Faculty supervise second and third year residents responding to overnight and weekend phone calls from patients without access to clinical records	• All faculty and patients have 24/7 remote access to EMR, including vaccination history and test results. • Faculty order follow-up appointments at time of visit so that staff schedule patients to see that provider at subsequent visits. All staff have access to EMR to view patients’ visit histories and schedule visits with relevant providers to promote continuity of care. • Faculty and residents utilize an EMR template that was instituted to document follow-up of overnight and weekend phone calls and ensure key elements (e.g. medications, chronic conditions, allergies, etc.) are reviewed.
**3. Population Health Management/ Knowing and Managing Your Patients**	
• Ad hoc reports about clinical quality metrics rely on administrative data, manual chart reviews, and/or publicly available datasets.	• Faculty review pre-defined clinical quality metrics that are tracked regularly for specific populations (e.g., asthma, ADHD, lead screening) during division-wide meetings. • Clinical decision-support tools implemented to support faculty in identifying patients with persistent asthma who are overdue for controller medication renewal, regular health maintenance visits, and vaccinations. • Nursing intake includes assessment of patients’ health literacy and cultural needs (e.g., preferred language) at least once annually and key elements of social history (e.g., financial stressors, housing instability) at annual health maintenance visits. Faculty and all other team members are able to view these intakes at any time.
**4. Care Management and Support**	
• Faculty collaborate with other healthcare personnel (e.g., social worker, nutritionist, etc.) for care coordination as needed.	• Faculty contribute to create patient-education materials and care plans for 0–6 month old infants, asthmatic and obese patients that are given to patients during check-out. • Scheduling staff run reports to identify patients who are overdue for specific care services (e.g., regular health maintenance visits, asthma management visits, and lead/hemoglobin screening) and contact patients to schedule visits to address these care needs.
**5. Care Coordination and Care Transitions**	
• Faculty collaborate with practice staff to assist families with care coordination and transitions as needed.	• Faculty offer enriched medical home service (i.e. home visitation by a trained community health worker and/or social worker support) to patients who are at-risk for poor health outcomes. • Practice staff run reports to identify outstanding orders (e.g., specialist referrals, lab tests, etc.) and contact patients to address these care needs. • When the practice is notified that patients have visited the ED/urgent care, practice staff call families to offer a follow-up appointment.
**6. Performance Measurement and Quality Improvement (QI)**	
• Ad hoc reports about clinical quality metrics rely on administrative data, manual chart reviews, and/or publicly available datasets.	• All faculty, clinical nursing leads, and office managers trained in the Plan-Do-Study-Act model. • All faculty participate in reviewing QI data regularly and some mentor residents’ QI projects and/or student scholarly projects in this area.

**Table 2:** Resident education: Strategies addressing PCMH principles pre- and post-transformation

Former State	Current State
**1. Team-Based Care**	
• Residents assigned to same practice site for entire duration of residency training. • 100 % dedicated time in outpatient setting during general ambulatory rotation. • Residents collaborate ad hoc with nurse and patient-care technicians during continuity clinic patient care sessions.	• Arrangement of schedules to allow for weekly resident clinic at specific continuity site with the same preceptor for the duration of their residency. • Staff schedule follow-up appointments with the same resident/preceptor pair whenever possible. • Residents mentored by faculty to lead daily huddles with nursing staff to proactively plan care and review pre-visit planning information during continuity clinic patient care sessions.
**2. Patient-Centered Access**	
• Primary management of overnight and weekend phone calls by second and third year residents under the supervision of an attending physician.	• 24/7 remote access to EMR for overnight and weekend calls transformation. • Institution of a process leveraging an EMR template for PCPs to follow up on overnight and weekend phone calls.
**3. Population Health Management/ Knowing and Managing Your Patients**	
• None	• Institution of a primary care track that includes 1–2 months dedicated to continuity clinic and outpatient primary care. • Faculty role model culturally competent practice skills to residents through exposure to a diverse patient population. • Nursing intake includes assessment of patients’ health literacy and cultural needs (e.g., preferred language) at least once annually and key elements of social history (e.g., financial stressors, housing instability) at annual health maintenance visits. Faculty and all other team members are able to view these intakes at any time.
**4. Care Management and Support**	
• Obtaining first-hand experience in the navigation and utilization of community resources during community medicine rotation.	• Identification of high risk patients to offer proactive care management. • Accompany trained community health workers on home visits and/or social worker consultation provided as an enriched medical home service. • Residents mentored by faculty participate in the creation of patient-education materials and care plans for 0–6 month old, asthmatic and obese patients.
**5. Care Coordination and Care Transitions**	
• Collaboration with non-physician staff such as social worker, nutritionist, etc. for care coordination. • Enriched medical home service offered to patients at risk for poor health outcomes.	• Institution of a formal sign-out procedure for graduating residents to handoff more complicated patients from their panel to incoming interns. • Creation of a resident lab pool for tracking consultations and outstanding lab/imaging test results.
**6. Performance Measurement and Quality Improvement**	
• Training in the Plan-Do-Study-Act model.	• Refined structure of clinical practice for residents to participate in quality improvement projects. • Residents participate in departmental quality assurance meetings and high reliability units.

**Table 3:** Medical student education: Strategies addressing PCMH principles pre- and post-transformation

Former Curricular Activity	Current State in Pre-Clinical Training	Current State in Clinical Training
**1. Team-Based Care**		
• Periodic team-based activities were integrated across the medical student curriculum.	• Team based learning case studies integrated into each pre-clinical course. • Team-based standardized patient encounters in all system-based courses, which include group history taking, examination, diagnosis and treatment.	• Clinical students participate in weekly “Family Centered Rounds” with a wide variety of healthcare professionals and their patient’s family to gather information and plan next steps. • Students are required to complete “translational pillars” in between clerkships in which groups work together to solve clinical problems.
**2. Patient-Centered Access**		
• Foundations in Clinical Practice course provided occasional lectures by patient speakers.	• “Meet the Patient” seminars to increase student exposure to the patient perspective. • Periodic one-on-one standardized patient encounters with a focus on patient care, integrated with feedback from the patient, preceptors, and self-auditing using interactive video capture.	• Students work in both in-patient and out-patient centers that are geographically dispersed in order to facilitate sociodemographic understanding. • Students rotating through the newborn nursery are tasked with extensive parental education to provide experience in patient instruction.
**3. Population Health Management/ Knowing and Managing Your Patients**		
• Students were introduced to basic concepts within epidemiology and public health in the Foundations in Clinical Practice course.	• “Themes in Medical Education” (TIME) course in the first and second year with structured interactive learning activities focused on epidemiology as well as systemic, state, and community support programs.	• In collaboration with institutional initiatives, students are afforded experiential opportunities to learn about critical public health issues in their local communities.
**4. Care Management and Support**		
• Education in care management and support was reserved for clinical students during “discharge rounds”.	• Standardized patient encounters focused on gauging patient health literacy and delivering patient education during the TIME course.	• Students in the Internal Medicine clerkship participate in weekly “multi-disciplinary care management” rounds. • Students are assessed on their ability to be active participants on patient rounds to encourage care management competency.
**5. Care Coordination and Care Transitions**		
• Students gained skills in care coordination and care transitions through active participation in clinical clerkships.	• Students gain exposure to multi-disciplinary care through visits to varied community health sites through the TIME course- during which students meet social workers, dietitians, nurses, and other healthcare team members.	• Phase III students are evaluated heavily on their ability to transition from reporter/interpreters of information to managers and educators.
**6. Performance Measurement and Quality Improvement**		
• Students discussed performance measurement and quality improvement in unstructured activities with both clinical and pre-clinical preceptors.	• Activities targeting hospital quality improvement during the TIME course. • Patient-led discussions surrounding care improvement and critique during the TIME course.	• Clinical students participate in structured chart reviews to assess practice performance. • Students are required to complete a one-time evaluation of medical school tenets about quality improvement measures and a medical error protocol.

## Results

### Team-based care

Our faculty retreat served as the venue to articulate our overarching goal to maximize face-to-face time between physicians and patients by empowering practice team members to function at ‘top of license’. Policies specifying the functional roles and responsibilities of each team member were implemented to standardize expectations across ambulatory practice sites and are now reviewed annually under the leadership of the division chief in collaboration with the medical director, practice administrator, and ambulatory nursing supervisor. Daily 5–10 min huddles led by faculty with the participation of residents reinforced respective roles initially and residents now lead daily huddles for their continuity sessions. Continuity of care with residents was supported by administrative procedures to schedule follow-up appointments for patients with the same resident-preceptor dyad whenever possible and by redesigning resident schedules. Furthermore, implementation of customized EMR order sets for routine health supervision visits by age per Bright Futures guidelines [20] include ordering the next routine appointment so that the front desk reception staff are required to schedule the next appointment when the family departs (i.e. ‘checks out’). The new medical school curriculum incorporated teamwork during the pre-clinical and clinical years. Upon matriculation to our institution, students are placed into groups of 6–8, working together throughout each phase of their education to complete a wide range of learning activities, including team-based standardized patient encounters. During the clinical years, students participate in “family centered rounds” on their Pediatrics clerkship with 1–4 students assigned to each clinical team. In these sessions, students work with the entire healthcare team and the patient’s own family to present the patient’s case and plan next steps.

### Patient-centered access

We implemented standardized scheduling templates that include three same-day-sick appointments per clinical session. This enabled patients to have emergent access to physicians that are most familiar with their care. The implementation of the EMR in conjunction with PCMH practice transformation further improved access in multiple ways. Attending physicians and residents gained 24/7 remote access to patients’ health records. A phone call documentation template was implemented; this template automatically retrieves important patient information (e.g., allergies, medications, etc.) from the EMR and prompts residents to complete a structured summary note of the call, including the final disposition, that is reviewed the following day by the practice team to ensure closed-loop communication. As required by NCQA, our site managers now audit 1 week of calls annually to assess compliance and have found no need for corrective action. In the medical school, an introduction to the patient perspective became an explicit focus during the pre-clinical training via the required Themes in Medical Education (TIME) course. As an example, students were given the opportunity to hear directly from a wide variety of patients about their experiences in the healthcare system via “Meet the Patient” seminars followed by debriefing sessions in small groups facilitated by faculty [21].

### Population health management/ Knowing and managing your patients

Faculty began using data extracted from the EMR to assess population-based metrics and support improvement efforts. For example, asthma management tools with discrete data elements were built into the EMR to allow assessment of Asthma Action Plan and Asthma Control Tool completion in comparison to pre-specified targets based on evidence-based guidelines. As required by NCQA, annual chart reviews assess utilization of these tools and have consistently shown > 80 % utilization for eligible patients. Our residency leadership team created a primary care track option for residents to have the opportunity to perform a 2 to 4-week ambulatory rotation at the continuity practice site of their choice. This furthered residents’ understanding of the patient demographics and health needs in their community by allowing them to work with populations in settings that differ from their usual continuity practice site. Additionally, residents now complete an 8-week community and advocacy rotation in which they engaged with various social welfare systems (e.g., family court, public education) to gain an intimate understanding of the community they serve while being introduced to established organizations. In the medical school, epidemiology and public health curricula in the “Foundations of Medical Practice Course” were reinforced by required visits to community-based healthcare organizations. Students used public health data to isolate a particular local healthcare issue and then are tasked with designing programs that work towards ameliorating that concern. Furthermore, through the TIME course, students were counseled on ways to encourage patients to access community-based resources. In particular, students are asked to use public health data to isolate a particular health care issue affecting our county and then tasked with designing programs that work towards ameliorating the selected public health concern. Most recently, our institution’s participation in New York State’s Delivery System Reform Incentive Program [22] was utilized to innovate programs from which students and faculty physicians can learn about the public health issues specific to Suffolk County and the patient population seeking care at our academic medical center.

### Care management and support

We implemented clinical care recommendations for faculty to identify and refer patients broadly deemed to be ‘at risk’ for poor health outcomes to the nationally recognized Keeping Families Healthy program [23, 24]. The program was a free, voluntary, enriched medical home service, in which trained community health workers provided support by making home visits complemented by remote phone/text follow-ups until participating families achieved self-sufficiency in following clinical care recommendations. Referring providers received summaries in the EMR after every home visit to review and revise in real-time, if needed. Residents also participated in 2–3 home visits with the program’s community health workers and were taught ways to identify and refer ‘at-risk’ patients. Additionally, resident clinical care teams at each practice site were responsible for identifying high-risk medically complex patients during the daily huddle to provide proactive care management, such as scheduling frequent office visits to monitor chronic health conditions or writing letters of medical necessity. Subsequently, a social worker was hired not only to provide care management for the highest risk patients but also to provide brief mental health interventions and serve as liaison for patients referred to psychiatric services. In the medical school, pre-clinical students received practical education in care management through a weekly exercise in the TIME course. Each week, students were assigned a care management task related to a condition or life-stage that is being discussed in the course at the time, such as informing a mother of her newborn child’s congenital disorder and recommending a course of action. Then, in a videotaped encounter with a standardized patient, students gauged patient health literacy, engagement, and understanding of the subject.

### Care coordination and care transitions

We created policies to facilitate communication between faculty and patients about orders for referrals and clinical tests. Under these policies, nursing staff were responsible for identifying all outstanding orders, communicating with families at least twice to encourage completion, and notifying providers if these orders were not completed so that the provider can determine best next steps (e.g., phone call by provider, defer discussion to next office visit, etc.). Furthermore, these policies ensured that consultation notes from subspecialists within our healthcare system were received in a timely way. As per NCQA requirements, annual audits of outstanding order reports have confirmed adherence to this process. Among the residents, care coordination was deeply integrated into the educational curriculum and practice workflow through regular collaboration between residents and co-located non-physician staff (e.g., site managers, social workers, nutritionists). Seamless care transitions were ensured through a sign-out procedure for graduating residents to formally handoff medically complex patients from their panel to incoming residents. In the medical school, pre-clinical students gained ample experience with care coordination and care transitions through the TIME course. In each TIME week, students had the opportunity to visit a variety of care locations and meet with various healthcare professionals in order to cultivate an appreciation for the complexity of coordinated care. During their clinical clerkships, medical students are heavily assessed on their abilities to coordinate care.

### Performance measurement and quality improvement (QI)

An ambulatory interdisciplinary leadership infrastructure and process were established to address this standard. Specifically, physician leads and administrative site managers at each ambulatory practice site met regularly with the division chief and medical director for primary care to review QI data and discuss best next steps. We also shared QI data division-wide at faculty meetings held every other month (see Supplemental Data Files 1, 2, 3). This infrastructure enabled open lines of inter-professional communication, real-time review of data, and refinement of interventions. The implementation of the QI standard provided residents opportunities to spearhead work on ambulatory projects under the mentorship of faculty. Results from these QI initiatives were regularly presented at departmental meetings as well as national conferences. At the medical school, pre-clinical students learned about the complexities of providing care through a number of TIME course exercises. Clinical students were expected to complete a one-time evaluation of hospital quality improvement measures and a medical error protocol in addition to having many first-hand learning opportunities during their clerkships.

## Discussion

In this paper, we describe our institution’s efforts to implement and sustain PCMH transformation educational initiatives concurrently across multiple levels of the healthcare provider hierarchy, from medical students to residents and medical school faculty. PCMH practice requires a shift in mental models across all healthcare workforce professionals involved in patient care [25] Our multi-pronged approach can be replicated and/or adapted by other AMCs striving to implement PCMH efficiently by leveraging the unique strengths of academic institutions that have influence across multiple educational stages of healthcare providers. Across the country, trends in hospital philosophies and medical education are pivoting towards an emphasis on patient-centered care and community conscious approaches. Given the continuing focus on population health management and the transition to value-based healthcare, we believe other AMCs and healthcare systems may leverage similar opportunities to implement multi-tiered educational and transformational efforts.

The use of a vertical integration approach employed the natural hierarchy in our academic medical center to achieve PMCH education at all levels (i.e. encouraging faculty transition to patient-centered rounds translated to resident and medical student involvement in these rounds and their subsequent education). Specifically, opportunities for changes in the medical school and residency curriculum were leveraged in order to guide a focus on increasing endorsement of and proficiency in the six PCMH tenets [26]. Community-based programs, such as Keeping Families Healthy, were integrated into the curriculum for residents, medical students, and faculty. Lastly, the comprehensive EMR system used in the medical school, hospital, and affiliated ambulatory clinics was used to facilitate improvements in care transitions, management, and QI.

As expected, we encountered multiple challenges during our PCMH implementation efforts including constraints of time, stakeholder resistance to change, and access barriers to technology. We aimed to overcome these efforts by utilizing change management principles that draw upon organizational behavior literature focused on people, processes, and technology [27, 28]. In particular, the simultaneous implementation of PCMH model across multiple geographically dispersed sites and an EMR within a very short timeframe of 18 months required careful and consistent planning. Initially, obtaining buy-in from key stakeholders in leadership and clinical practice was a key focus and required multiple face-to-face meetings to convey the importance, short-term financial benefits, and potential long-term benefits for institutional strategic positioning and planning. In order to gain buy-in from stakeholders and limit resistance to change, leaders from all constituencies were recruited to become PCMH champions. Ensuring that processes put into place leveraged the already strong infrastructure of site-based personnel to embed new ways of working in teams to enable each team member to work at ‘top-of-license’ was a key guiding principle throughout the implementation. Lastly, proactively shaping EMR implementation with recommended processes for documentation by all members of the workforce was a critical component to support our PCMH transformation efforts. Finding ways to customize our EMR to enable key quality reporting and support efficient clinical documentation (e.g., developing an asthma action plan that pulls key information from data already in the system) required close collaboration with our institution’s information technology leadership and staff. To ensure that all employees were comfortable with the use of this new technology, and therefore able to participate in the PCMH framework, EMR trainings were held at multiple times (e.g., morning, afternoon, evening), in multiple formats, and with numerous supports available. Overall, we utilized these change management tactics to create an enduring cultural change, allowing the PCMH transformation to be sustainable[29].

There are some limitations to our work. One limitation of our report is the focus on physicians and physicians-in-training. In practice, our approach required the inclusion of other healthcare professionals to be successful—particularly for implementation of care coordination and care transition principles [1, 30]. However, describing our approach for nursing and ancillary personnel in detail were beyond the scope of this particular report. Another limitation of the generalizability of our work is the fact that our institution is an academic medical center and a state-affiliated medical school whose primary mission includes education and, as such, has access to educational and public funds (e.g., graduate medical education, state-based grants) that other health care systems may not have. Nevertheless, many of the changes implemented in conjunction with the PCMH model were inexpensive, practical, and efficient (e.g. scheduling and rounding style changes, partnering with existing community organizations) [31]. We believe some of these changes can be implemented – or adapted- in many care settings. Notably, our report also does did not measure educational outcomes (e.g., level of learner competence, faculty competence) or experiences of trained staff, which are important topics to study [32]. A final limitation to this approach is that our report describes the implementation approach based on our experience with a single hospital and 9 ambulatory clinical sites in our system. However, our institution serves a diverse patient population that is generally reflective of our national population and has grown to encompass several hospitals and 15 ambulatory clinical sites. We are now leveraging our proven track record in championing successful implementation, our established standardized processes, and shared electronic medical record platform to extend our approach throughout our growing system.

AMCs are uniquely positioned to educate and influence the physician workforce across this continuum and engage in interdisciplinary teamwork which is critical for the successful implementation of PCMH [33]. Ultimately, AMCs education and implementation efforts focused on PCMH principles influence not only the quality of healthcare delivery for patients and families today but also the principles espoused by the next generation of physicians caring for patients and families in the future [34]. Many of the changes implemented in conjunction with the PCMH model were inexpensive, practical, and efficient (e.g., scheduling and rounding style changes, partnering with existing community organizations). In addition, the multi-faceted innovative transformation described here that began in 2014–2015, created the foundation for a deeply rooted patient-centered culture that has been successfully sustained and leveraged to achieve recognition on annual basis. Our AMC now includes 15 sites with over 200 medical staff that have integrated the PCMH standards into routine care so that our practices efficiently achieved PCMH recognition during the 2020 cycle and are well-positioned to achieve annual recognition.

## Supplementary Information

**Additional file 1:**

**Additional file 2:**

**Additional file 3:**

## Data Availability

Data sharing is not applicable to this article as no datasets were generated or analyzed for the purpose of this study. As described in the manuscript, data were generated for submission to NCQA as required for PCMH recognition and are presented in aggregate in this manuscript.

## Abbreviations

AMC: Academic Medical Center
EMR: Electronic Medical Record
NCQA: National Committee for Quality Assurance
PCMH: Patient Centered Medical Home
QI: Quality Improvement
TIME: Themes in Medical Education
