# Supplementary material for: Healthcare workforce transformation: implementing patient-centered medical home standards in an academic medical center
Source: BMC Med Educ. 2021 Jun 3;21:313. doi: 10.1186/s12909-021-02775-9 (PMC8173877; doi:10.1186/s12909-021-02775-9)
Supplement: Supplementary file 1 — Additional file 1: [file 12909_2021_2775_MOESM1_ESM.docx]

|  | **Pre-transformation** | **Jan-Mar 2018** | **Oct-Dec 2020** |
| --- | --- | --- | --- |
| Children’s Immunization Status^1^ | 63/120 | 1122/1353 | 1463/1680 |
|  | 52.5% | 82.9% | 87.1% |
| HPV^2^ | Not available | 3065/8114 | 2766/4466 |
|  |  | 37.8% | 61.9% |
| Well Child Visits for 3-6 year olds^3^ | 1000/1500 | 5109/6973 | 2985/3014 |
|  | 66.7% | 73.3% | 99.0% |
| Asthma Medication^4^ | Not available | 218/378 | 462/749 |
|  |  | 57.7% | 61.7% |
| Pediatric Symptom Checklist screening^5^ | Not available | Not available | 1490/1632 |
|  |  |  | 91.3% |

**Clinical Performance Measures for Pediatric Practices:**

**Children’s Immunization Status [1]:**Data pre-transformation was obtained via manual chart reviews in which 30 charts were randomly selected from 4 sites. Subsequently, our EMR reporting system implemented NQF0038 as a certified electronic clinical quality measure (eCQM).

Denominator: Patients who turned 2 years old during the report period.
Numerator: Patients who were in the denominator and had four diphtheria, tetanus and acellular pertussis (DtaP); three polio (IPV); one measles, mumps and rubella (MMR); three H influenza type B (HiB); three hepatitis B (HepB); one chicken pox (VZV); four pneumococcal conjugate (PCV); two hepatitis A (HepA); two or three rotavirus (RV); and two influenza (flu) vaccines by their second birthday.
 **^2^HPV:**
This report’s source prior to Q4 2019 was our administrative billing systems. Subsequently, our integrated data platform entitled HealtheAnalytics was implemented (merges EMR data with administrative billing data, undergoes multiple validation checks, and includes provider attribution logic).

Definition in 2018:

Denominator: Patients who turn 11-15 years old (inclusive) and had at least one well child visit (using ICD10 codes: Z00.121, Z00.128, Z00.129) at any of the pediatric primary care sites during the measurement period.
Numerator: Patients who are in the denominator and received at least one HPV vaccine during the measurement period (note: the HPV vaccine could be given at any visit).

Definition in 2020:

Denominator: Patients who were >=9 years and <27 years and had at least one routine health supervision visit (using ICD10 codes: Z00.121, Z00.128, Z00.129) at any of the pediatric primary care sites during the measurement period. Notably, pediatric primary care practices see patients until age <22 years.
Numerator: Patients who were in the denominator and received at least one HPV vaccine during the measurement period (note: the HPV vaccine could be given at any visit).
 **^3^Well Child Visit 3-6 years old:**

This report’s source prior to Q4 2019 was our administrative billing systems. Subsequently, our integrated data platform entitled HealtheAnalytics [2] was implemented (merges EMR data with administrative billing data, undergoes multiple validation checks, and includes provider attribution logic).

Denominator: Patients who were 3-6 years old as of the last date in the reporting period.
Numerator: Patients who were in the denominator and had at least one well child visit during the 15-month look-back period.

**^4^Asthma Medication:**
Prior to 2019, reports required the provider list to be manually updated in our EMR.

Denominator: Patients who had persistent asthma during the reporting period and were seen for any office visit during the quarter.
Numerator: Patients who were in the denominator and received a controller asthma medication during 6 month look-back period.

**^5^Pediatric Symptom Checklist screening:**
Screening results were entered into discrete fields in the EMR beginning in late 2018.

Denominator: Patients of age >=11 years and <18 years who were seen for a well visit at any of the pediatric primary care sites during the reporting time period.
Numerator: Patients who are in the denominator and had a completed pediatric symptom checklist (PSC or Y-PSC as appropriate) screening within 12 months period of time of the visit date.

1. Center for Medicare & Medicaid Services. *2014 Clinical Quality Measures (CQMs) Pediatric Recommended Core Measures*. 2014. Accessed on 3.23.2021 <https://www.cms.gov/Regulations-and-Guidance/Legislation/EHRIncentivePrograms/Downloads/2014_CQM_PrediatricRecommended_CoreSetTable.pdf>

2. Cerner Corporation. *HealthAnalytics.* Accessed on 3.23.2021 <https://www.cerner.com/pages/cerner-healtheanalytics>
