# Supplementary material for: Healthcare workforce transformation: implementing patient-centered medical home standards in an academic medical center
Source: BMC Med Educ. 2021 Jun 3;21:313. doi: 10.1186/s12909-021-02775-9 (PMC8173877; doi:10.1186/s12909-021-02775-9)
Supplement: Supplementary file 2 — Additional file 2: [file 12909_2021_2775_MOESM2_ESM.docx]

**Clinical Performance Measures for Family Medicine Practices:**

| \|  \| **Pre-transformation (2013)** \| **July – Dec 2017** \| **Jul-Sept 2020** \| \| --- \| --- \| --- \| --- \| \| **Flu Shots for Adults ages 50-64** \| 9/20 \| Not reported \| Not reported \| \| 45.0% \|  \|  \| \| **Adult BMI assessment** \| 13/20 \| Not reported \| Not reported \| \| 65.0% \|  \|  \| \| **Comprehensive Diabetes Care: LDL-C Screening** \| 18/20 \| Not reported \| Not reported \| \| 90.0% \|  \|  \| \| **Use of spirometry testing in the assessment and diagnosis of COPD** \| 9/20 \| Not reported \| Not reported \| \| 45.0% \|  \|  \| \| **Medical assistance with Smoking and tobacco cessation: Advising Tobacco users to quit** \| 17/20 \| Not reported \| Not reported \| \| 85.0% \|  \|  \| \| **Pneumococcal vaccination in adults 19-64 years old** \| Not available \| 159/4558 \| 1069/2201 \| \| 3.5% \| 48.6% \| \| **Colorectal cancer screening; patients age 50 to 75** \| Not available \| 2169/4197 \| 2286/3562 \| \| 51.7% \| 64.2% \| \| **Diabetes Mellitus types 1 and 2 with HbA1c less than 9%** \| Not available \| 790/1416 \| 730/1391 \| \| 55.8% \| 52.5% \| \| **PHQ2 screening** \| Not available \| 1088/1349 \| 1025/1226 \| \| 80.7% \| 83.6% \| |
| --- | --- | --- | --- | --- | --- | --- | --- | --- | --- | --- | --- | --- | --- | --- | --- | --- | --- | --- | --- | --- | --- | --- | --- | --- | --- | --- | --- | --- | --- | --- | --- | --- | --- | --- | --- | --- | --- | --- | --- | --- | --- | --- | --- | --- | --- | --- | --- | --- | --- | --- | --- | --- | --- | --- | --- | --- | --- | --- | --- | --- | --- | --- | --- |

Notes: Data pre-transformation was obtained via manual chart reviews from one site as specified for each measure. Subsequently, our organization’s integrated data platform entitled HealtheAnalytics^1^ was implemented in 2017 (merges EMR data with administrative billing data, undergoes multiple validation checks, and includes provider attribution logic) and this facilitated a transition to select different measures that included data from both practice sites for the purposes of maintaining patient-centered medical home recognition.

**Flu Shots for Adults ages 50-64:**Data pre-transformation was obtained via manual chart reviews in which 20 charts were randomly selected from one site.

Denominator: Patients of 50-64 years of age seen during the project reporting time period.
Numerator: Number of patients who received influenza vaccination during the project reporting time period.
 **Adult BMI Assessment:**Data pre-transformation was obtained via manual chart reviews in which 20 charts were randomly selected from one site.
Denominator: Patients of 18-74 years of age seen at the practice site during the project reporting time period.
Numerator: Number of patients who of 18-74 years of age and have their BMI documented during the project reporting time period.

**Comprehensive diabetes Care: LDL-C Screening**Data pre-transformation was obtained via manual chart reviews in which 20 charts were randomly selected from one site.
Denominator: Patients with diabetes seen at this site during the project reporting time period.
Numerator: Number of patents with LCL-C < 130 mg/dl testing results during the project reporting time period.

**Use of spirometry testing in the assessment and diagnosis of COPD**Data pre-transformation was obtained via manual chart reviews in which 20 charts were randomly selected from one site.
Denominator: Patients with COPD seen at this site during the project reporting time period.
Numerator: Number of COPD patents who have documented spirometry testing during the project reporting time period

**Medical Assistance with Smoking and Tobacco Cessation: Advising Tobacco Users to Quit**Data pre-transformation was obtained via manual chart reviews in which 20 charts were randomly selected from one site.
Denominator: Patients who smoke seen at this site during the project reporting time period.
Numerator: Number of smokers counseled to quit during the project reporting time period.

**Pneumococcal vaccination in adults 19-64 years old:**Denominator: The number of patients ages ≥19 years and ≤ 64 years who were seen at practice during the reporting period.
Numerator: The number of patients in the denominator who had pneumococcal vaccination.

**Colorectal cancer screening; patients age 50 to 75 years:**
Denominator: Patients ages ≥ 50 years and ≤ 75 years who were seen at the practice during the reporting period.
Numerator: The number of patients in the denominator who have received colorectal cancer screening.

**Diabetes Mellitus types 1 and/or 2 with HbA1c less than 9%:**Denominator: The number of patients age ≥ 18 years with Diabetes Mellitus types1 and/or 2 and were seen at the practice during the reporting period.
Numerator: The number of patients in the denominator whose HbA1c is less than 9%.

**PHQ2 screening:**
Denominator: The number of patients age ≥ 18 years who has at least one health maintenance visit at the practice during the reporting period.
Numerator: The number of patients in the denominator who had at least one PHQ2 screening during the reporting time period.

1. Cerner Corporation. *HealthAnalytics.* Accessed on 3.23.2021 <https://www.cerner.com/pages/cerner-healtheanalytics>
