# Supplementary material for: Healthcare workforce transformation: implementing patient-centered medical home standards in an academic medical center
Source: BMC Med Educ. 2021 Jun 3;21:313. doi: 10.1186/s12909-021-02775-9 (PMC8173877; doi:10.1186/s12909-021-02775-9)
Supplement: Supplementary file 3 — Additional file 3: [file 12909_2021_2775_MOESM3_ESM.docx]

**Clinical Performance Measures for General Medicine Practices:**

| \|  \| **Pre-transformation (CY 2013)** \| **Jan – June 2017** \| **Oct-Dec 2020** \| \| --- \| --- \| --- \| --- \| \| **Preventive Care and Screening: Tobacco Use: Screening and Cessation Intervention** \| 2176/3211 \| Not reported \| Not reported \| \| 67.8% \|  \|  \| \| **Controlling high blood pressure** \| 6842/7704 \| Not reported \| Not reported \| \| 88.8% \|  \|  \| \| **Adult weight screening and follow up** \| 3315/9073 \| Not reported \| Not reported \| \| 36.5% \|  \|  \| \| **Diabetes: hemoglobin A1c poor control** \| 1422/1693 \| Not reported \| Not reported \| \| 84.0% \|  \|  \| \| **Diabetes: Blood pressure management** \| 1275/1930 \| Not reported \| Not reported \| \| 66.1% \|  \|  \| \| **Pneumonia vaccination of patients 65 and older** \| Not available \| 3359/4144 \| 3942/4531 \| \| 81.1% \| 87.0% \| \| **Colorectal cancer screening; patients age 50 to 75** \| Not available \| 3285/5193 \| 3595/4885 \| \| 63.3% \| 73.6% \| \| **Diabetes Mellitus types 1 and/or 2 with HbA1c less than 9%** \| Not available \| 1509/1952 \| 2024/2568 \| \| 77.31% \| 78.8% \| \| **PHQ2 screening** \| Not available \| Not available \| 1874/2872 \| \| 65.3% \| |
| --- | --- | --- | --- | --- | --- | --- | --- | --- | --- | --- | --- | --- | --- | --- | --- | --- | --- | --- | --- | --- | --- | --- | --- | --- | --- | --- | --- | --- | --- | --- | --- | --- | --- | --- | --- | --- | --- | --- | --- | --- | --- | --- | --- | --- | --- | --- | --- | --- | --- | --- | --- | --- | --- | --- | --- | --- | --- | --- | --- | --- | --- | --- |

Notes: Our organization’s integrated data platform entitled HealtheAnalytics was implemented in 2017 (merges EMR data with administrative billing data, undergoes multiple validation checks, and includes provider attribution logic) and this facilitated a transition to select different measures for the purposes of maintaining patient-centered medical home recognition and meeting meaningful use measures^1^. Notably, a new location practice location was opened during 2017.

**Preventive Care and Screening: Tobacco Use: Screening and Cessation Intervention:**Denominator: Patients age ≥ 18 years who have been seen for at least 2 office visits during the measurement reporting time period.
Numerator: The number of patients in the denominator who were queried about tobacco use one or more times within the preceding 24 months. **Controlling high blood pressure:**Denominator: The number of patients who are ≥ 18 years and have had at least two face-to-face encounters with the provider during the reporting period and have a diagnosis of hypertension in the summary active problems field or encounter assessment field.
Numerator: The number of patients in the denominator who have at least one blood pressure reading performed and recorded in the vital signs chart section during the reporting period.

**Adult weight screening and follow up:**Denominator: The number of patients between the ages of 18 years and 64 years of age by the start of the reporting period and have at least one face-to-face encounter with the provider during the reporting period.
Numerator: The number of patients in the denominator that have a normal BMI (BMI>18 or <25) OR an abnormal BMI (BMI>=25 or <18.5) recorded in the Vital Signs chart section (BMI must be recorded in the six months prior to the encounter date or during the reporting period).

**Diabetes: hemoglobin A1c poor control:**
Denominator: The number of patients that were between 18 and 74 years of age by the start of the reporting period and have at least one face-to-face encounter with the provider during the reporting period and have a diagnosis of Diabetes (recorded in the patient's Summary Active Problems field OR encounter Assessment fields.
Numerator: The number of patients in the denominator that have had an HbA1c >9% documented using summary Interventions field or the encounter plan field.

**Diabetes: Blood pressure management:**
Denominator: The number of patients that were between 18 and 75 years old at the start of the reporting period have at least one face-to-face encounter with the provider during the reporting period, and have a diagnosis of Diabetes must be recorded in the patient's Summary Active Problems field or Encounter Assessment.
Numerator: The number of patients in the denominator that have a blood pressure reading documented during the reporting period, in the Vital Signs chart section. If the patients’ blood pressure reading was <140/90.

**Pneumonia vaccination of patients 65 and older:**
Denominator: The number of patients age ≥ 65 years who were seen at practice during the reporting period.
Numerator: The number of patients in the denominator who have received pneumococcal vaccination.

**Colorectal cancer screening; patients age 50 to 75:**
Denominator: The number of patients age ≥ 50 and ≤ 75 years who were seen at practice during the reporting period.
Numerator: The number of patients in the denominator who received colorectal cancer screening.

**Diabetes Mellitus types 1 and 2 with HbA1c less than 9%:**Denominator: The number of patients age ≥ 18 years who have Diabetes Mellitus (types 1 or 2) and were seen at practice during the reporting period.
Numerator: The number of patients in the denominator whose HbA1c is less than 9%.

**PHQ2 screening:**
Denominator: The number of patients age ≥ 18 years who had at least one health maintenance visit at practice in the preceding 12 months.
Numerator: The number of patients in the denominator who had a PHQ2 screening during the reporting time period.

1. Center for Medicare and Medicaid Services. 2017 Modified Stage 2 Program Requirements for Providers Attesting to their State’s Medicaid EHR Incentive Program. Accessed on 5.5.2021 <https://www.cms.gov/Regulations-and-Guidance/Legislation/EHRIncentivePrograms/Stage2MedicaidModified_Require>
